# Seroprevalence and Risk Factors of Chikungunya Virus Infection in Mayotte, Indian Ocean, 2005-2006: A Population-Based Survey

**DOI:** 10.1371/journal.pone.0003066

**Published:** 2008-08-26

**Authors:** Daouda Sissoko, Amrat Moendandze, Denis Malvy, Claude Giry, Khaled Ezzedine, Jean Louis Solet, Vincent Pierre

**Affiliations:** 1 Institut de veille sanitaire, Cellule Interrégionale d'Epidémiologie Réunion et Mayotte, Saint-Denis, La Réunion, France; 2 Direction des Affaires Sanitaires et Sociales de Mayotte, Mamoudzou, France; 3 Service de Médecine Interne et des Maladies Tropicales, Hôpital Saint André, Centre Hospitalier Régional et Universitaire, Bordeaux, France; 4 Institut de Médecine Tropicale et EA 3677 Faculté de Médecine, Université de Bordeaux, Bordeaux, France; 5 Centre Hospitalier de Mayotte, Laboratoire de biologie, Mamoudzou, France; Columbia University, United States of America

## Abstract

**Background:**

Since 2006, Chikungunya virus (CHIKV) has re-emerged as an important pathogen of global concern. However, individual and household factors associated with the acquisition and the magnitude of clinically silent CHIKV infections remain poorly understood. In this present study, we aimed to investigate the seroprevalence, estimate the proportion of symptomatic illness and identify the risk factors for CHIKV infection in the primo-exposed population of Mayotte.

**Methods/ Principal Findings:**

We conducted a household-based cross sectional serosurvey in Mayotte in November and December 2006 using complex multistage cluster sampling. To produce the results representative of the island population aged 2 years or older, sample data were adjusted with sample weights. Explanatory and multiple logistic regression analyses were performed to investigate associations between CHIKV infection seropositivity (presence of IgM and/or IgG to CHIKV by enzyme-linked immunoabsorbent assay) and risk factors. A total of 1154 individuals were analyzed. The overall seroprevalence of CHIKV infection was 37·2% (95% CI = 33·9–40·5), 318 (72·3%) of the seropositive participants reported symptoms consistent with a CHIKV infection during the epidemic period. Risk factors for CHIKV seropositivity among adults (aged 15 years and older) were male gender, low socioeconomic index, schooling ≤6 years and living in makeshift housing.

**Conclusions:**

Our findings indicate that roughly one out of four CHIKV infections is asymptomatic. Conditions associated with poverty may be considered as critical in CHIKV acquisition. Thus, these conditions should be taken into account in the development of future prevention strategies of CHIKV disease.

## Introduction

Chikungunya virus (CHIKV) is an arthropod-borne virus primarily transmitted to humans through the bite of infected mosquitoes of the genus *Aedes* [1]. Since its first recognition in 1952–1953 [2], both sporadic cases and major epidemics of CHIKV disease have been reported in Africa [3], India [4], South-East Asia and the Western Pacific [5], [6]. Classically, CHIKV fever appears suddenly as a non-specific febrile illness often associated with pronounced polyarthralgia and a skin rash. Typically, these clinical manifestations resolve within 7 days [7], [8]. Nonetheless, its characteristic clinical symptom, polyarthralgia, may persist as disabling and long-lasting joint pain [9], with a serious social and economic impact on both the individual and the affected communities [10]. Historically, CHIKV disease was long considered to be a non-fatal disease and systemic involvement or haemorrhagic manifestations were infrequently reported [11], [12]. However, several worrying facets of CHIKV infection have recently been revealed in areas with access to sophisticated medical infrastructure in a number of well-documented epidemics. These include peripartum mother-to-infant transmission [13], severe neurological involvement [14] and mortality [15], [16].

In 2005–2006, epidemics of CHIKV disease of unprecedented magnitude occurred in the islands of the South-West Indian Ocean [17]–[19]. Subsequently, the virus's rapid geographic spread led to a large CHIKV disease epidemic in India in 2006–2007 [20]. More surprisingly, an epidemic of CHIKV disease, arising from an imported index case from India, was reported for the first time in the Northern Hemisphere in 2007 in Italy [21], where replication-competent vectors have established themselves since the early 1990s [22]. As a result, CHIKV will probably continue to expand into other new temperate areas where competent vectors reside [23], [24].

Both the widening geographical spread of CHIKV epidemics and increased awareness of the potential severity of infection have drawn attention to this re-emerging pathological agent as a public health threat of global relevance. The majority of published research into CHIKV epidemiology has concentrated on vector density and ecological and climatic conditions [1], [25]. In contrast, other epidemiological aspects of CHIKV infection, such as the determinants of the individual risk of CHIKV infection in established epidemics, remain sparsely documented.

The Indian Ocean island of Mayotte provides an opportunity to evaluate the primary epidemiology and clinical magnitude of CHIKV infections. The presence of Chikungunya virus was detected in Mayotte for the first time at the end of the rainy season in April 2005. This was followed by a large scale epidemic in the following rainy season of 2006 and, in May 2006, nearly 26% of inhabitants were estimated to be infected with CHIKV [19]. Mayotte is an overseas French-administrated territory located in the Comoros archipelago, South-Western Indian Ocean. According to the 2002 census, the estimated population in 2006 was 175 000 with a density of 468 inhabitants/km^2^. The climate is characterised by a temperate dry season from May to October, and a hot and rainy season from November/ December to April. The population is primarily of African origin. A flow of immigrants from the neighbouring Comoro Islands accounts for approximately 35% of the population of Mayotte. Self-subsistence agriculture and fishing or the informal market are the principal sources of employment. The quality of housing varies greatly according to socio-economic status.

In order to address several poorly understood epidemiological features of CHIKV, we conducted a household-based survey of serological and clinical magnitude of CHIKV infections in the population of Mayotte Island exposed for the first time to CHIKV. The objectives of the study were to estimate the proportion of symptomatic infection and to identify the most important individual and household risk factors for the infection.

## Methods

### Study Population

A household-based cross sectional survey including determination of serological status for CHIKV antibodies was carried out in November and December 2006, within nine months of the peak of the epidemic. Considering the evolution of the epidemic, two-wave-phenomenon was observed. The first wave centred in the North-eastern part of the island started in mid-April 2005, peaked in May and waned by July 2005. As the environmental temperature increased during the following hot and rainy season, the second and main wave that encompassed the entire island, started in January 2006, peaked during the months of March and April, and thereafter declined. By late July 2006, the epidemic was completely controlled. Overall, the attack rates of the epidemic were estimated at least 1.6% out of 175 000 inhabitants in 2005 (≈2800 CHIKV cases) and 26% in 2006 (≈45000 CHIKV cases) according to per-epidemic surveys [19].

Participants aged 2 years or older were selected using a multiple-stage sampling procedure [26]. A target sample size of at least 850 individuals was determined, assuming a prevalence rate of 25%, a type I error of 0.05, a precision of 5% and a design effect of 3.0, in order to take into account the cluster sampling design [27].

In a first step, 40 of the 400 clusters provided by the 2002 census blocks were randomly sampled proportionally to population size. In a second step, 7 to 12 households (defined as a group of individuals sleeping or eating together) were selected within each cluster using a stereotyped walk from a randomly selected start point. Finally, in each selected household, all adults (aged 15 years and older) and one individual aged less than 15 years were invited to participate in the survey [28]. Individuals aged between 2–14 years were thus sampled relatively less frequently than older individuals, given that 43% of the total population of Mayotte is aged 0–14 years. In order to optimise coverage, household visits were also made during weekends and holidays. Moreover, when eligible members of the house were absent at the time of the initial visit, interviewers could make two additional return visits in order to include temporarily absent individuals. However, individuals who could not be reached after a maximum of three visits were not included in the recruitment procedure. Participation in the study was entirely voluntary and a signed consent was obtained from each participant.

In the interest of representativity, the sample was weighted according to sociodemographic characteristics in order to represent the total Mayotte population and to account for potential oversampling and nonresponse. Population weights were obtained from the Regional Bureau of the French National Institute for Statistics and Economic Studies using the 2006 report of the 2002 population census.

### Data Collection

A pre-tested structured questionnaire was administered face-to-face in the home by local trained interviewers in Shimaori, the local language. The following categories of data were collected:

- Sociodemographic characteristics: age, gender, educational level, country of origin, years of residence in Mayotte, employment. Due to the broad age range of the study sample, some of these variables, namely educational level, country of origin, years of residence in Mayotte and employment status, were considered not to be relevant for the risk factor analysis in individuals less than 15 years of age.
- Housing and peridomestic environment characteristics: type of house construction, number of household residents, sanitary conditions, waste disposal, household facilities such as electricity, a source of drinking water, and vegetable garden. Information on housing characteristics and asset-ownership was obtained from a reference correspondent for the household, who was by default the oldest adult resident.
- Clinical characteristics: self-reported history of an acute febrile illness consistent with presumptive CHIKV fever and month and year of onset. For this purpose, we used a calendar of locally-important events to facilitate recall of dates**.** Whether a participant had a history of CHIKV fever was addressed by the question, “Have you ever had an acute febrile illness consistent with a possible diagnosis of CHIKV fever between February 1, 2005 and the date of the interview?” Individuals who responded ‘No’ or ‘Don't know’ were considered not to have a history of acute febrile illness. Participants who reported a potential history of CHIKV fever provided information on symptoms by free-recall which was then cross-checked against a list of common symptoms of CHIKV infection (for example, polyarthralgia) in the questionnaire. Parents or legal guardians were interviewed on these items for subjects under 15 years of age.

A six-point household asset index was used as a proxy of socio-economic status for adults. This index was constructed on the basis of availability of electricity (1 point), a flush toilet within the house (1 point), piped water source for drinking (1 point), and possession of a television set (1 point), radio (1 point), and refrigerator (1 point). These items were summed for each household, and the distribution of the household asset index score established for the total study sample. On the basis of the median score, the study sample was divided into two grades, namely low economic status (total score<median threshold) and medium to high socioeconomic status (total score≥median threshold).

A venous blood sample was obtained from each participant. Immediately after puncture, blood samples were stored 4–8°C and forwarded on the same day to the laboratory of Mayotte's Territorial Hospital, which was responsible for all serologic testing. Testing was performed according to the guidelines of the French National Reference Centre for Arboviruses and WHO Collaborating Centre for Haemorrhagic Fevers and Arboviruses, Pasteur Institute, Lyon, France, which provided the reagents. All serum samples were tested for specific anti-CHIKV immunoglobulin M (IgM) antibodies by IgM-capture enzyme-linked immunosorbent assay (MAC-ELISA) [29] and for specific IgG antibodies by ELISA [30]. Specimens with absorbance >3 standard-deviations above the mean absorbance of negative controls were considered as positive.

A CHIKV-positive individual was defined as a participant with IgG or IgM serum antibodies to CHIKV while a CHIKV-negative individual had no detectable serum antibodies to CHIKV.

Symptomatic CHIKV infection was defined as a CHIKV-positive individual who recalled having experienced acute febrile syndrome consistent with CHIKV fever during the 2005–2006 epidemics. Asymptomatic CHIKV infection was defined as a CHIKV-positive individual who did not report episodes of acute febrile syndrome during the epidemic period.

### Statistical methods

Data were double entered and corrected for data entry errors using EPIDATA® software version 3.0. (Epidata Association, Odense, Denmark). All analyses were performed with STATA® software version 9.0 (Stata Corporation, College Station, USA). We assessed crude prevalence estimates without weighting in order to avoid potential spurious percentage transformations.

In an explanatory bivariate analysis, we examined potential associations between CHIKV seropositivity and sociodemographic characteristics and housing features using population-adjusted weights to account for the survey design. Overall and subgroup-specific CHIKV seroprevalence rates were estimated and potential differences between subgroups evaluated using binomial survey-adjusted Wald chi-square tests. A probability level of less than 0.05 was considered statistically significant. In these analyses, primary sampling units (census area) were grouped into one of four possible regions of residence: North-East, North, Midwest and South.

In a following step, we investigated independent associations between explanatory and outcome variables while controlling for confounders in a multivariable logistic regression analysis restricted to adult subjects (≥15 years). We performed two separate logistic regression models with either sociodemographic or household variables as explanatory variables and CHIKV serological status as the dependant variable. This allowed the main independent variables for CHIKV infection occurrence to be identified in each model.

To build up multivariate models, all variables significantly associated with serological status at a probability threshold of ≤0.25 level in the previous explanatory analyses based on binomial survey-adjusted Wald chi-square tests were selected for input into the models.[31] Thirty six subjects were excluded from the model since data was missing for one or other of these independent input variables. Each model thus contained 852 observations corresponding to this number of adult subjects. Backward stepwise selection procedures were used to retain proposed variables in each final model. Concurrently, a two-by-two control procedure for interaction terms between explanatory variables was applied. The strength of the association between risk factors and CHIKV seropositivity in the multivariate analyses was estimated as odds ratios (OR) with 95% confidence intervals (95% CI). All OR excluding 1.0 were considered to be significant in each final model. In order to reflect association with outcome adequately, confidence intervals for OR estimates were calculated on the basis of a log transformation with the standard errors computed by the delta method that incorporated all sources of variation resulting from the sampling cluster design of the study [32].

### Ethical considerations

Pre-notification and information about the survey was given through the local media use and personal contact with representatives of local authorities. During survey visits, interviewers introduced themselves and explained the objectives and all procedures to all potential interviewees in the selected households. A written consent form was obtained from adults or guardians of those individuals aged less than 18 years prior to inclusion. If the legal guardian was illiterate, signed consent was sought from another literate family member chosen by the guardian. Ethical review and approval were granted by the Human Subjects Protection Committee of Bordeaux University Hospital Centre, France, in compliance with French regulations on the protection of human subjects. A subject number was attributed to each participant and used to label blood samples. All other personal information was removed before data entry on database.

## Results

### Survey participation

**Figure 1:**
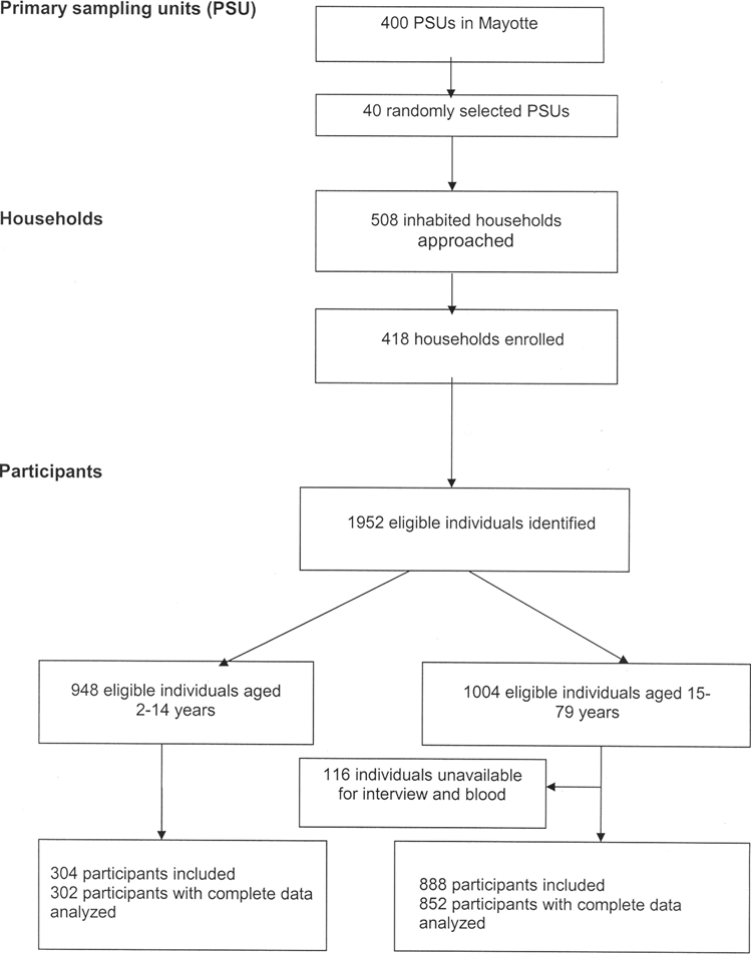
Study participation profile.

Study interviewers approached a total of 508 inhabited households and obtained permission to enter the house at 418 (82%) of these (Figure 1). In these households, 1952 eligible individuals were identified, of whom 948 were aged between 2–14 years and 1004 were adults (≥15 years). Of these, 304 subjects in the 2–14 year age group and 888 subjects in the adult age group consented to be interviewed and provide a blood sample. The remaining 116 adults (11·5%) were unavailable after three household visits. Two participants in the 2–14 year age group did not provide sufficient serum to determine serological status and 36 adults with incomplete data were thus excluded from the analysis. The total study sample analyzed thus consisted of 1154 persons.

### Population and household characteristics

The final study sample consisted of 655 (58·8%) females and 499 males. The sociodemographic characteristics of the sample are presented in Table 1. Of the 852 adults, 52·3% were born in Mayotte, 40·9% elsewhere in the Comoros Islands and 6·8% were from another origin. Of those 406 adults who were born outside Mayotte, the median time of residence in Mayotte was 9 years. Most of the adults had a low education level, finishing their schooling before the end of primary school (80%).

**Table 1:** Weighted seroprevalence of anti-CHIK virus antibodies according to demographic characteristics in individuals aged ≥2 years, Mayotte, 2005–2006.

Characteristic	N	Weighted prevalence, % (95% Confidence interval)	p-value*
All participants (2–79 years)	1154	37.2 (33.9–40.5)	
Gender			0·03
Male	499	40.6 (32.6–48.7)	
Female	655	33.8 (23.8–43.9)	
Age group, years			
2–14	302	33.2 (24.2–42.2)	0·33
15–24	294	39.3 (27.2–51.5)	
25–34	193	41.7 (29.7–53.7)	
35–44	169	40.2 (29.7–50.8)	
45–54	107	26.9 (15.1–38.8)	
≥55	89	36.6 (23.3–49.8)	
Birthplace†			0·0005
Mayotte	446	28.4 (21.2–35.7)	
Other Comoro island	349	52.8 (40.6–65.1)	
Other place	57	24.3 (9.8–38.8)	
Length of education)†			0·003
0–6 y	679	42.1 (32.3–51,9)	
>6 y	173	25.7 (16.4–35.1)	
Occupation			0.035
Employed	189	28.6 (19.9–37.3)	
Schooled	179	34.2 (22.1–46.2)	
Unemployed	484	44.8 (33.5–56.2)	
Length of residence in Mayotte‡			0.70
0–2 y	45	41.7 (24.0–59.4)	
3–9 y	166	50.9 (33.9–68.0)	
≥10 y	195	48.9 (36.2–61.6)	

^*^ Probabilities were calculated using an adjusted Wald *X^2^* test within subgroups; ^†^ Only for adults ; ^‡^ For those born outside of Mayotte.

Comparison of the sociodemographic distribution of the study sample closely matched that of the general Mayotte population as described in the 2002 census report. However, men were somewhat under-represented compared to women (43·2% vs. 50·2% in the 2002 census) and individuals in the 15–24 age group were slightly over-represented (25·5% vs. 20·2% in census 2002). These minor differences were corrected by appropriate weighting for the analysis of seroprevalence and risk factors.

Of the 418 households, most of them were constructed of concrete (n = 256; 61·2%); 327 (78·2%) had piped water but only 149 (35·7%) were equipped with flush toilets. The household asset index was rated as low for 121 households (29%). In particular, 98% of the houses were not equipped with window or door screens and; in 77·8%, domestic waste was disposed of outside the compound.

### Seroprevalence of anti-Chikungunya virus antibodies

The overall weighted seroprevalence rate for anti-CHIKV antibodies in the study population was 37·2% (95% CI 33.9–40.5). The weighted prevalence of specific IgM and IgG antibodies was 18·1% and 37·2% respectively. Significant differences in seroprevalence were observed according to gender (40·6% for men and 33·8% for women, p = 0·03), but not for age, although subjects in the 2–14 and 45–54 year age groups appeared to be less infected. Associations were also observed between the presence of CHIKV antibodies and birthplace, length of education and occupation status (Table 1).

Furthermore, weighted seroprevalence rates varied from 3·6% to 86·7% between the 40 primary sampling units. The lowest seroprevalence rate was observed in individuals living in the Southern region (5·4%) and the highest rate in those living in the Northern region (45·1%). Finally, seroprevalence varied substantially according to housing standards, especially with respect to construction type, household size and asset index (Table 2).

**Table 2:** Weighted seroprevalence of anti-CHIK virus antibodies according to household features in individuals aged ≥2 years, Mayotte, 2005–2006.

Characteristics	N	Weighted prevalence, % (95% Confidence interval)	p-value*
Construction type			
Concrete	708	30.2 (21.9–38.4)	
Adobe and stone	345	44.2 (31.1–57.4)	0·058
Makeshift	101	65.6 (47.6–83.6)	
Household size (no. of members)			
1–2	101	35.1 (24.4–45.8)	
3–4	276	46.7 (36.6–56.9)	0·054
≥5	777	34.3 (24.6–44.1)	
Region of residence			
Northeast	417	43.2 (26.9–59.5)	
North	255	45.1 (29.2–60.9)	
Midwest	303	38.2 (26.6–49.8)	
South	179	5.4 (1.7–9.8)	
Asset index			
Below the median threshold	288	61.2 (45.9–76.5)	0·0009
At or over the median threshold	866	31.6 (23.8–39.4)	

* Probabilities were calculated using an adjusted Wald *X^2^* test within subgroups.

### Prevalence of symptomatic CHIKV infection

Among 440 persons with CHIKV antibodies, 318 (72·3%) reported occurrence of febrile symptoms compatible with a CHIKV infection during the epidemic, compared with 107 of 714 (15%) seronegative participants (P<0·0001). The proportion of individuals with symptomatic CHIKV infection increased with age from 63% in the 2–14 year age group, 67% in the 15–24 year age group, 75% in the 25–34 year age group, 87% in the 25–34 year age group, 83% in the 45–54 year age group and finally 73% in the ≥55 year age group (*X^2^* for trend = 9·85, P<0·001).

The crude odds ratio for the relative risk of symptom presentation in individuals seropositive for CHIKV antibodies was of 14·8 and the proportion of subjects whose clinical symptoms could be attributed to CHIKV infection during the 2005–2006 epidemics was estimated to be 93·2% (Table 3).

**Table 3:** Comparison of self-reported symptoms consistent with CHIKV infection according to the presence of any CHIKV-specific antibody, Mayotte, 2005–2006.

	CHIK antibodies pos* CHIK antibodies neg† No of participants (%)	
Self-reported CHIK symptoms		
Yes	318 (72.3)	107 (15)
No	122 (27.7)	607 (85)
Total	440	714
Symptoms in persons with CHIK antibodies	Crude odds ratio 14.8 (11.03–19.8)	
	Attributable fraction 93.2% (90.9%–95.0%)	

* Positive.

† Negative.

In the 318 participants with symptomatic laboratory-confirmed CHIKV infection, the recalled date of symptom presentation ranged from February 2005 to September 2006. The most frequently reported symptoms were polyarthralgia (N = 314; 99%), muscular pain (N = 296; 93%), backache (N = 273; 86%) and abrupt onset fever (N = 269; 85%). Age ≥25 years was a significant predictor of symptomatic infection with a crude odds ratio of 2·14 (95% CI 1·39–3·29).

### Individual and household factors associated with the presence of anti-Chikungunya virus antibodies

The final multivariate model for sociodemographic characteristics identified significant associations with CHIKV seropositivity for being born in an other island of the Comoros archipelago (OR 2.62; 95% CI 1.72–4.00), male gender (OR 1·45; 95% CI 1·07–1·95), and short schooling duration (OR 1·68; 95% CI 1·06–2·67). A non-significant association was also observed for “being unemployed or housewife” (Table 4).

**Table 4:** Adjusted odds-ratios for the presence of any CHIK virus antibody according to demographic characteristics of individuals aged ≥15 years, Mayotte, 2005–2006.

Characteristics	Adjusted odds ratio (95% Confidence interval)
Gender	
Male	1.45 (1.07–1.95)
Female	1.00
Birthplace	
Mayotte	1.00
Other Comoros island	2.62 (1.72–4.00)
Other	1.04 (0.49–2.19)
Length of schooling, y	
0–6	1.68 (1.06–2.67)
≥6	1.00
Occupation	
Worker/employed	1.00
Schooled	1.58 (0.87–2.84)
Unemployed/Housewife	1.61 (0.97–2.67)

In the final multivariate model for household-related features (Table 5), associations with CHIKV seropositivity were observed for a low household asset index (OR 1·65; 95% CI 1·21–2·25) and for makeshift housing construction (OR 3·08; 95% CI 1·09–8·65). In the final models, no pairwise interactions among variables were statistically significant.

**Table 5:** Adjusted odds-ratios for the presence of any CHIK virus antibody according to household features of individuals aged ≥15 years, Mayotte, 2005–2006.

Characteristics	Adjusted odds ratio (95% Confidence interval)
Construction type	
Concrete	1.00
Adobe and stone	1.20 (0.67–2.16)
Makeshift	3.08 (1.09–8.65)
Household size (no. of members)	
1–2	1.00
3–4	1.65 (0.89–3.06)
≥**5**	1.08 (0.64–1.84)
Asset index*	
Below the median threshold	1.65 (1.21–2.25)
At or over the median threshold	1.00

* This asset index included following the parameters: electricity, flush toilet within the household, piped water as source of drinking water, possession of a television set, radio, refrigerator.

## Discussion

This large-scale, population-based survey is one of the first to investigate risk factors for CHIKV infection and on the rate of clinical manifestations in a newly exposed community. During the massive CHIKV epidemic in the South-western Indian Ocean Islands in 2005–2006, 37·2% of individuals living in Mayotte were infected. It is likely that low-level transmission continued throughout the dry and temperate months of 2006 resulting in a higher seroprevalence than the rate of 26% previously reported in May 2006 [19]. Additionally, we found evidence for a high proportion of symptomatic CHIKV infection even though younger individuals (2 to 24 years) were significantly less likely to exhibit symptomatic infection. This difference may be related to host determinants and needs further elicitation of the mechanisms underlying symptom development.

Another striking feature of this survey relates to the observed regional variation in CHIKV seropositivity, with southern rural districts being less affected than the north-eastern and northern urban districts. Indeed, the North of Mayotte is considered to be an area owing higher degree of urbanisation than the South. It can be speculated that this geographical variation may reflect differences in urbanisation and population densities between these areas. We thus postulate that CHIKV transmission is more effective in densely-inhabited urban and peri-urban areas in Mayotte since humans are thought to be the only reservoir of the virus during epidemics and this reservoir is thus larger in urban areas [33], [34].

Furthermore, all classes of age had been infected to a similar extent. This finding supports the hypothesis that background immunity for CHIKV infection was virtually absent within the population of Mayotte, in agreement with the low 1·9% seroprevalence in pregnant women (1.9%) observed in a previous survey in October 2005 [19]. Surprisingly, we found that CHIKV seroprevalence was higher in men than in women. This finding is inconsistent with previous reports from the Comoros and Reunion Islands [17], [18] which indicate that women are more prone to CHIKV infection. Similar discrepancies have been reported with dengue virus which shares common vectors and shows relatively similar transmission dynamics to CHIKV infection [35]. In some reports, men appeared to be more susceptible to dengue infection whereas in others, women were more affected, and no gender differences were noted in others [36]–[38]. This inconsistency may relate to gender differences in exposure to infection due to community-specific habits, customs or behaviours.

The study was specifically designed to document and evaluate the influence of the diversity of socioeconomic status and housing conditions on the acquisition of CHIKV infection. Over the last decade, the island of Mayotte has been experiencing since several years uncontrolled population flux due to its relatively wealthy status compared to other islands in the Comoros archipelago. These data are the first to demonstrate that poor living conditions are strongly associated with high risk for CHIKV infection. In particular, makeshift housing and a low household asset index were associated with higher CHIKV seroprevalence rates. These observations are consistent with previous findings from studies of dengue fever in the USA, where seroprevalence rates varied with socio-economic status between neighbouring areas in Texas- Mexico border [39], [40]. Nonetheless, short duration of schooling and immigration from other islands of the Comoros archipelago were also associated with an increased risk of CHIKV infection. These two variables are likely to be related to poverty. Many individuals of Comorian origin are often illegal immigrants with rough living conditions and low incomes. Such conditions are mainly encountered in populous urban or peri-urban areas where people are more vulnerable to infectious diseases. It should be noted that no significant association was observed between CHIKV seropositivity and length of residence among immigrants from the other Comoros Islands. For this reason, our findings do not support the hypothesis that the elevated seroprevalence in this group could be explained by imported new CHIKV cases linked to migratory fluxes.

The lack of association between CHIKV seroprevalence and the peridomestic environment (water recipients, tyres, inappropriate waste disposal) was unexpected, since these are considered as sources of *Aedes* spp mosquitoes. This finding may primarily be explained by control measures accompanied by large media coverage put in place during the epidemic, and public health education campaigns promoting physical elimination or alteration of breeding sources and peridomestic spraying of chemical insecticides. Since these activities were ongoing at the time of this study, it is probable that less human-made breeding sites for mosquitoes were available than would be usual. For this reason, it appears that peridomestic environment as described in our study may not reflect completely pre-epidemic exposure to mosquitoes. Our study design did not include entomological investigations, which would require a different data collection approach beyond the objective of this work. The most suitable design for collecting entomological parameters would be a comprehensive, prospective, longitudinal study capable of identifying local entomological determinants and transmission patterns.

The occurrence of the 2005–2006 episode of CHIKV infection in Mayotte took place within the context of a long-lasting regional-wide CHIKV fever epidemic. Chikungunya virus seroprevalence rates in Mayotte were similar to those documented in Reunion Island, but lower than those reported in the Comoros Republic and Lamu, Kenya, (63% and 75% respectively) [17], [41]. These differences may reflect both vector capacities and measures taken in response to the epidemic. Indeed, *Ae. Aegypti*, incriminated as the principal vector involved in the Comoros outbreak [42], is recognised as the most competent vector for CHIKV transmission, whereas *Ae. albopictus*, which was the principal *Aedes* species incriminated both in Reunion Island and in Mayotte, is usually considered to be a secondary vector [43]. Surprisingly, *Ae. albopictus* has been shown to be a very efficient vector in the context of the later phases of the epidemic in the above areas [44], [45]. Nonetheless, the relative importance and efficiency of the different mosquito vectors of CHIKV in any of these large epidemics remains unclear. Moreover, Reunion and Mayotte Islands, which are French-administered territories, are likely to have benefited from higher resource allocation to control the epidemic. Consequently, the huge public health information campaigns aimed at aggressive mosquito extermination and larval control activities taken in these territories probably contributed to limit the infected vector population and ultimately reduce CHIKV transmission to humans.

Nonetheless, some findings of this study should be interpreted with caution. Firstly, the history of symptomatic presumptive CHIKV fever depended on participant-self report of an acute febrile condition that occurred during the epidemic period. Our subsequent estimate of the proportion of symptomatic CHIKV infection was based on these self-reported retrospective data. Therefore, occurrence of bias linked to misclassification or recall might lead to error in the estimate of the proportion of infected individuals with clinically manifest disease. However, in an attempt to validate answers obtained from the survey, each participant was asked to provide details on any apparent symptoms alleged to be simultaneous with the supposed CHIKV infection. We found that 99% of seropositive symptomatic participants recalled experiencing polyarthralgia which is the most noticeable characteristic symptom of CHIKV fever. We assume this reliable feature is less vulnerable to recall bias compared to other common symptoms such as fever. Moreover, differentiation of CHIKV fever from other tropical diseases presentation on a clinical basis remains challenging. To the best of our knowledge, dengue fever is a very rare condition in Mayotte and until now, there is no reported circulation of other arthrogenic arboviruses in this setting. The most likely conflicting differential diagnosis would be malaria attack, and to some extent leptospirosis. Thus misclassification based on febrile illness cannot be definitely excluded. Nevertheless, such potential misclassification should not account for an important impact on our estimated prevalence of symptomatic confirmed CHIKV fever. Another limitation of the study relates to the validity of the classification of socio-economic status. We combined household asset ownership into a composite index of socioeconomic status. Constructing such an index remains very complex in the absence of consensus on the choice of parameters to incorporate and their corresponding weight [46]. In this respect, we focused on data reflecting socioeconomic variables that were pertinent to the households studied, although the relevance of the assets chosen may clearly vary from country to country. Consequently, we used a standard index which is pertinent in this particular setting and cannot be directly applied to other contexts. Despite this limitation, the impact of the socioeconomic surrogate index on the prevalence of CHIKV infection was significant, and it should be considered as an important independent determinant of infection when planning further prevention strategies.

Our findings have important public health and social implications. Firstly, the recent introduction of CHIKV in Mayotte is clearly assumed to have produced partial herd immunity in the population. Nonetheless, the occurrence of further epidemics in the future clearly remains plausible. Early detection of such phenomena emphasises the need for sustainable laboratory-based, active surveillance programmes in Mayotte and elsewhere. Secondly, these results also highlight the need to prevent CHIKV infection and other vector-borne infections transmitted by established local vectors. Such prevention efforts should be designed taking into account reliable data on risks of transmission. Here, we have provided early evidence on the contributing role of conditions related to poverty in CHIKV infection. As a result, policy makers and health authorities should take into account the reduction of social inequalities and investment in housing rehabilitation as well as vector control and public education as components of prevention strategies both in Mayotte and probably in other tropical countries where wide socio-economic disparities between populations exist.
